# Some Experiences in the Treatment of Arterial Injuries
*A paper read to the Bristol Medico-Chirurgical Society on Wednesday, April 14th.


**Published:** 1948

**Authors:** J. J. Mason Brown

**Affiliations:** Edinburgh


### SOME EXPERIENCES IN THE TREATMENT OF ARTERIAL INJURIES *

BY

J. J. Mason Brown, *O.B.E.*, M.B., Ch.B.

*Edinburgh.*

---

Arterial injuries are encountered infrequently in civilian surgical practice and for that very reason may give rise to difficulty in diagnosis. The various types of arterial injury and the complications which may follow them are summarized in the following table:—

| | | | |
|---|---|---|---|
| Arterial spasm | | | |
| Arterial contusion | thrombosis → peripheral embolus | | |
| | secondary haemorrhage | | |
| | delayed traumatic aneurysm | | |
| Arterial wounds | Complete division (little or no bleeding) | | |
| | Partial division—profuse bleeding | external | |
| | | internal | arterial hæmatoma |
| | | | false aneurysm |
| Wounds of artery and companion vein | varicose aneurysm | | |
| | aneurysmal varix | | |

### The effects of Arterial Injury on the Peripheral Circulation

Following arterial injury the peripheral blood-flow is maintained by the damaged artery and by the collateral circulation. The blood-flow through the main vessel is interrupted in complete transection of the artery, in severe arterial spasm and in thrombosis following arterial contusion. Blood-flow through the affected artery continues in mild arterial spasm, minor contusions without thrombosis, arterial hæmatoma and in aneurysmal varix. The continuing blood-flow is less than normal but is often sufficient to maintain the nutrition of the part. Primary ligation in such cases will place the limb at the mercy of a precarious collateral circulation which has had no time to develop.

Diminution of the circulation through the main artery is shown

* A paper read to the Bristol Medico-Chirurgical Society on Wednesday, April 14th.

by weakness or loss of the peripheral pulses but this sign alone is not evidence of dangerous peripheral ischæmia, the signs of which are pallor or cyanosis of the digits, coldness of the limb, loss of function and loss of sensibility of the glove or stocking type. In the absence of the peripheral pulses the limb is certain to survive if active digital movement is present, if the skin of the digits shows some warmth, some sensation and a not unnatural colour (Mustard 1946).

*The immediate Treatment of Blood Vessel injuries.* External haemorrhage makes immediate resuscitation and surgical control of the bleeding imperative. Subcutaneous injuries and those associated with minor wounds and little or no external haemorrhage are more difficult to diagnose and to treat. The diagnosis depends on the presence or absence of a swelling in the line of the artery, auscultation over the vessel and the state of the peripheral pulses. In the absence of swelling and murmur, loss of the peripheral pulses may result from arterial spasm, arterial thrombosis or from complete transection of the vessel. In the early stages of the traumatic arterial hæmatoma the swelling does not pulsate but a faint systolic murmur is audible over it. In all cases the examination is completed by assessing the viability of the limb (*vide supra*).

It is well known that ischæmia of sudden onset is followed by such severe damage to the tissues that permanent disablement will follow in those cases in which gangrene does not occur. Ischæmia of gradual onset allows time for the development of the collateral circulation. In injuries in which the damaged vessel still permits the passage of blood and in which clinical examination reveals no evidence of severe ischæmia conservative measures may be followed by the survival of a useful limb. Non-intervention deprives the patient of the benefits of primary wound excision: but I have seen many patients none the worse for minor or through-and-through wounds which had received no primary surgical treatment. The results of primary ligature of such critical vessels as the popliteal are so disastrous that conservative measures are justifiable provided that they do not endanger the patient's life in the attempt to save his limb.

CASES WITHOUT LOCAL SWELLING OR MURMUR BUT WITH SIGNS OF DANGEROUS PERIPHERAL ISCHÆMIA require immediate operation, the treatment depending on the lesion displayed.

*Arterial Spasm.* Wounds of the soft tissues are excised and the artery is exposed but is handled and freed as little as possible to avoid increasing the spasm. In severe spasm it is difficult to identify the artery because of its string-like appearance. Under direct vision any fracture is reduced and loose fragments of bone likely to compress the vessel are removed. Moist heat is then applied to the vessel in the hope that the spasm will pass off. Local injections and periarterial sympathectomy are of no value and excision of the segment removes the main circulatory channel in the hope that it will also relieve the spasm of the collateral branches. The experimental work of Barnes and Trueta (1942) has demonstrated that lumbar ganglionectomy releases the spasm of the collateral branches but has little effect on that of the main artery. While ganglionectomy is a formidable addition to the primary surgical treatment Warren (1946) believes it to be the best method of treatment. In our experience the spasm has always passed off following exploration and it seems reasonable to try the effects of paravertebral block before proceeding to sympathectomy. At the conclusion of the local exploration only the skin is sutured to avoid tension and compression while in cases with gross swelling the wound may initially be left widely open, to be closed later by delayed suture. If the injury is a closed one it is justifiable to reduce the fracture by manipulation and await results before exploration is carried out.

*Arterial Contusion without Thrombosis.* In these cases it is unlikely that there will be peripheral ischæmia in the absence of associated arterial spasm : the diagnosis of arterial injury may not be made until the lesion is found during excision of the wound. The artery is still playing an important part in maintaining the peripheral circulation. Excision of the damaged segment has been advocated in order to prevent the dangerous sequelae of arterial contusion, but this will necessarily result in the abrupt interruption of the blood-flow. Maybury (1944) has suggested that the surgeon should imitate nature by interposing a barrier of muscle or fascia between the artery and the skin surface. Should thrombosis of the artery follow, the cutting off of the main blood-flow will be less abrupt: while in other cases the development of a traumatic aneurysm will allow the blood-flow through the artery to continue and operation on the aneurysm can be carried out when the collateral circulation is well established. Excision of the damaged segment and its immediate replacement by a vein graft is a more modern alternative.

*Arterial Contusion and Thrombosis.* The pulseless artery is playing no part in the peripheral circulation and the affected segment should be excised to prevent the complications of the lesion and to release the reflex spasm of the collateral vessels. Restoration of the artery by a venous graft should only be considered if the limb has been ischæmic for less than ten hours.

*Transection of the Artery.* Ligation will be necessary unless the conditions are favourable for arterial suture or venous grafting. Ligation must always be carried out above and below the lesion and the vessel is then divided between the ligatures. The best site for the application of the ligatures is still not established. Too few cases of primary ligation by Holman's (1944) or Lambert Rogers's (1945) methods are available for comparison with those of direct ligation.

Simultaneous ligation of the companion vein was advised by Makins on the evidence afforded by the arterial injuries of the first World War. The results of experimental work have not been conclusive. De Bakey and Simeone (1946) evaluated its effect on the arterial wounds in the American forces in the recent war: They conclude that simultaneous vein ligation makes no difference to the results of primary ligation of the main peripheral arteries. When the collateral circulation is well established concomitant ligation of the vein may be followed by the disabling venous obstruction syndrome described by Patterson Ross (1946).

The effect of sympathectomy on the results of primary ligation is difficult to evaluate. In many of the war injuries in which sympathectomy was carried out the operation was done too late in the vain hope of saving already ischæmic limbs. To be of value sympathectomy should be carried out before, or at the same time as, the ligation of the artery. There is, however, some evidence that sympathetic block was of value after primary ligation.

CASES WITH LOCAL SWELLING AND/OR MURMUR

*Traumatic Arterial Hæmatoma.* As already mentioned the blood-flow through the affected vessel continues and it was our practice to delay operation until the collateral circulation was established unless complications made earlier operation imperative. These complications are external haemorrhage, increasing peripheral ischæmia due to the pressure of the rapidly enlarging hæmatoma on the collateral vessels, infection in wounds in close proximity to the hæmatoma and severe pain. Emergency operation was required in twelve out of our total of seventy-eight cases. When the use of a tourniquet is impossible these cases may be very difficult for the tissues are solidly matted together. The damaged artery is ligated in most instances but in some arterial suture is possible.

*Traumatic Aneurysm.* The diagnosis is usually easy but the vessel affected may be difficult to determine in areas such as the root of the neck. There is a pulsatile swelling close to the line of the artery and a systolic murmur is audible over it. The murmur is conducted distally to a limited extent and the peripheral pulses may be diminished or delayed. Operation should be delayed until the collateral circulation is established. Sympathectomy was not carried out as a pre-operative measure in our cases and seems to be unnecessary with a fully established collateral blood-flow. When a tourniquet cannot be employed the operation is planned to gain temporary control of the artery above and below the aneurysm. The sac is opened and its contents are evacuated. The interior is then inspected and the various openings into it are investigated. The orifices of the smaller branches entering the sac are occluded by suture and attention is then directed to the main artery. In most cases the artery is ligated from within the sac, while in others it is possible to carry out suture or aneurysmorrhaphy. In rare instances in our series of cases the sac was excised and the artery mobilized sufficiently for end-to-end suture to be carried out. The temporary control of the blood-flow is then relaxed and if hæmostasis is satisfactory the wound is closed. Removal of the sac is likely to damage some of the collateral vessels and perhaps nerves embedded in the wall.

*Varicose Aneurysm.* This lesion presents clinically as a pulsatile swelling in the line of the artery. There is usually a well-marked thrill over it and the murmur is louder and rougher and more continuous than in arterial lesions. In most cases the murmur is conducted widely. General circulatory changes may occur as in aneurysmal varix when there is a significant venous shunt. The operative treatment is similar to that employed in arterial aneurysms but is delayed for longer to give more time for the collateral circulation to develop. In addition to the artery the vein requires to be ligated or repaired.

*Aneurysmal Varix.* There is no, or only a slight local swelling, which is due to the dilatation of the vein. There is a well-marked thrill over the lesion which may be noticed by the patient when his limb comes into contact with a surface. A rasping, continuous, " machinery " murmur is loudest over the fistula or where the artery comes nearest to the surface and is widely conducted. The pulse is of the collapsing type, capillary pulsation is well marked and the veins are distended but may be obscured by oedema and are seldom seen to pulsate. The heart enlarges, the blood volume may increase and there is a mild polycythaemia and an increase in the total circulating hæmoglobin. Compression of the artery or vein proximal to the lesion results in a slowing of the pulse which returns more gradually to its previous rate on releasing the compression (Branham's phenomenon). On proximal compression there is also a rise in the diastolic pressure. The site of the fistula may be established by auscultation or, in some cases, by arteriography. Operation is carried out without the aid of a tourniquet, the artery being displayed proximal to the lesion and followed peripherally until the fistula is found. All branches close to the fistula are then occluded by temporary tape loops. In some cases it is possible to separate artery and vein and repair them by suture, in others the fistula may be ligated or transvenous suture may be carried out. In most cases, however, ligation is performed and it is essential to ligate the artery close to and proximal and distal to the lesion. Quadruple ligation is usually advocated but the possibility of the venous obstruction syndrome must be considered.

---

The author quoted cases illustrating various points and concluded with an analysis of his own cases of arterial injury.

### REFERENCES

Barnes, J. M., and Trueta, J. (1942), *Brit. J. Surg.*, *30*, 74.
De Bakey, M. E., and Simeone, F. A. (1946), *Ann. Surg.*, *123*, 534
Holman, E. (1944), *Surg. Gynae. Obstet.*, *78*, 275.
Maybury, B. C. (1944), *Brit. Med. Bull.*, *2*, 142.
Mustard, W. T. (1946), *Ann. Surg.*, *126*, 46.
Rogers, L. C. (1945), *Med. J. Aust.*, *1*, 517.
Ross, J. Paterson (1946), *Brit. Med. J.*, i, 1.
Warren, L. R. (1946), *Arch. Surg.*, *53*, 86.

